# Supplementary material for: Fibrosis growth factor 23 is a promoting factor for cardiac fibrosis in the presence of transforming growth factor-β1
Source: PLoS One. 2020 Apr 21;15(4):e0231905. doi: 10.1371/journal.pone.0231905 (PMC7173860; doi:10.1371/journal.pone.0231905)
Supplement: Attachment — Submitted filename: Response to Reviewers2.doc [file pone.0231905.s002.doc]

Susumu Minamisawa, MD, PhD

Department of Cell Physiology

The Jikei University School of Medicine

3-25-8 Nishi-Shimbashi, Minatoku

Tokyo 105-8461, Japan

Tel: +813 3433 1111 ext. 2220

Fax: +813 3433 1279

sminamis@jikei.ac.jp

April 2nd, 2020

Dr. Joerg Heber

Editor-in-Chief, ***PLoS One***

Dear Dr. Heber,

We are grateful for the opportunity to revise our manuscript (PONE-D-19-34439), titled “**Fibrosis growth factor 23 is a promoting factor for cardiac fibrosis in the presence of transforming growth factor-β1**” and for the reviewers’ helpful comments. We were delighted to learn that you and the reviewers consider our work to be important. We have responded to each of the reviewers’ comments in detail (please see our “Responses to the reviewers’ comments”), and have modified the manuscript accordingly.

The authors would like to sincerely thank you and the reviewers for your time and commitment. We believe that the revised version of our manuscript is much improved, and it is our hope that it will be deemed suitable for publication in your esteemed journal.

Best regards,

Susumu Minamisawa

**Point-by-point responses to the reviewers’ comments**

**Response to Reviewer 1:**

We thank Reviewer 1 for his/her thorough review of the manuscript. We have responded to Reviewer 1’s comment and have modified the manuscript accordingly. It is our hope that the revised version is now deemed acceptable.

*1. Type I Collagen should be upregulated with the EMT markers. Why was the Type I collagen not upregulated with alpha-SMA in the experiment? It is not consistent with your hypothesis.*

**Thank you for pointing out the important issue. We also think that collagen should be increased as alpha-SMA is up-regulated.** **Collagen has been reported to be increased early in response to stimuli and then be decreased (*see the references below). We measured the expression levels of mRNAs at 72 hours after stimuli and this might be too late to catch the changes in the type I collagen expression. We have mentioned this possibility in the Discussion section of the revised manuscript and would like to confirm this in our future research.**

***** **Smith RL, Lin J, Trindade MC, Shida J, Kajiyama G, Vu T, et al.** Time-dependent effects of intermittent hydrostatic pressure on articular chondrocyte type II collagen and aggrecan mRNA expression. .**J Rehabil Res Dev.** 2000;37(2):153-61.

*** Xiaodong Pan, Zhongpu Chen, Rong Huang, Yuyu Yao, and Genshan Ma.** Transforming Growth Factor β1 Induces the Expression of Collagen Type I by DNA Methylation in Cardiac Fibroblasts. **PLoS One.** 2013; 8(4): e60335. doi: 10.1371/journal.pone.0060335
